# Smartphone-Based Chemiluminescent Origami µPAD for the Rapid Assessment of Glucose Blood Levels

**DOI:** 10.3390/bios11100381

**Published:** 2021-10-09

**Authors:** Donato Calabria, Martina Zangheri, Ilaria Trozzi, Elisa Lazzarini, Andrea Pace, Mara Mirasoli, Massimo Guardigli

**Affiliations:** 1 Department of Chemistry “Giacomo Ciamician”, Alma Mater Studiorum-University of Bologna, Via Francesco Selmi 2, I-40126 Bologna, Italy; donato.calabria2@unibo.it (D.C.); martina.zangheri2@unibo.it (M.Z.); ilaria.trozzi2@unibo.it (I.T.); elisa.lazzarini6@unibo.it (E.L.); andrea.pace7@unibo.it (A.P.); mara.mirasoli@unibo.it (M.M.); 2 Interdepartmental Centre for Industrial Agrofood Research (CIRI AGRO), Alma Mater Studiorum-University of Bologna, Via Quinto Bucci 336, I-47521 Cesena, Italy; 3 Interdepartmental Centre for Industrial Research in Advanced Mechanical Engineering Applications and Materials Technology (CIRI MAM), Alma Mater Studiorum-University of Bologna, Viale Risorgimento 2, I-40136 Bologna, Italy; 4 Interdepartmental Centre for Industrial Research in Renewable Resources, Environment, Sea and Energy (CIRI FRAME), Alma Mater Studiorum-University of Bologna, Via Sant’Alberto 163, I-48123 Ravenna, Italy; 5 Interdepartmental Centre for Industrial Aerospace Research (CIRI AEROSPACE), Alma Mater Studiorum-University of Bologna, Via Baldassarre Canaccini 12, I-47121 Forlì, Italy

**Keywords:** blood analysis, chemiluminescence, biosensor, enzyme assay, glucose, hydrogen peroxide, origami, paper-based analytical devices, point of care, smartphone

## Abstract

Microfluidic paper analytical devices (µPADs) represent one of the most appealing trends in the development of simple and inexpensive analytical systems for diagnostic applications at the point of care (POC). Herein, we describe a smartphone-based origami µPAD for the quantitative determination of glucose in blood samples based on the glucose oxidase-catalyzed oxidation of glucose leading to hydrogen peroxide, which is then detected by means of the luminol/hexacyanoferrate(III) chemiluminescent (CL) system. By exploiting the foldable µPAD format, a two-step analytical procedure has been implemented. First, the diluted blood sample was added, and hydrogen peroxide was accumulated, then the biosensor was folded, and a transport buffer was added to bring hydrogen peroxide in contact with CL reagents, thus promoting the CL reaction. To enable POC applicability, the reagents required for the assay were preloaded in the µPAD so that no chemicals handling was required, and a 3D-printed portable device was developed for measuring the CL emission using the smartphone’s CMOS camera. The µPAD was stable for 30-day storage at room temperature and the assay, displaying a limit of detection of 10 µmol L^−1^, proved able to identify both hypoglycemic and hyperglycemic blood samples in less than 20 min.

## 1. Introduction

Biosensors that fulfill the ASSURED (affordable, sensitive, specific, user-friendly, rapid and robust, equipment-free, and deliverable to end-users) World Health Organization criteria have become widely accepted as the benchmark for an ideal test that can be used for diagnostic purposes at the point of care (POC). Therefore, substantial efforts have been made to reach this goal, although challenges remain. Recently, two additional criteria were proposed to yield the REASSURED concept, where R indicates real-time connectivity and E stands for ease of specimen collection and environmental friendliness [1].

In this context, one of the most appealing trends in the development of simple and low-cost analytical systems that can be easily employed by unskilled users is represented by microfluidic paper-based analytical devices (µPADs) [2,3,4,5,6]. These devices take advantage of several features offered by the paper support, which is an inexpensive and renewable material, easily machined and adaptable to mass manufacturing processes, and eco-friendly disposable by incineration. By exploiting fluids movement through the porous substrate by capillary action, µPADs do not need pumping systems and require only small sample and reagents volumes. In addition, paper patterning to produce hydrophilic channels delimited by hydrophobic barriers and dry reagents storage on the paper support allow complete analyses to be easily performed in a very compact analytical format. Furthermore, the three-dimensional (3D) µPADs, such as the foldable µPADs that exploit the origami principle (i.e., implementing flow and mixing control by folding/unfolding the paper device), enable the execution of complex multistep analytical protocols that require a series of sequential operations [7].

In recent years, smartphone-based biosensors have proven a powerful approach for facilitating the widespread application of biosensors at the POC and for pursuing the mobile health and precision medicine approaches [8]. Modern smartphones provide data processing and storage capabilities, wireless connectivity, and built-in CMOS cameras that can be used for the development of biosensors based on optical detection [9]. Among the various optical detection principles employed in smartphone-based µPADs, chemiluminescence (CL), i.e., the photon emission triggered by a chemical reaction, is particularly advantageous, owing to its high specificity and detectability and to its amenability to miniaturization [10,11,12]. Although several µPADs with CL detection have been proposed for a variety of applications [13], they often involve complex analytical protocols requiring sequential addition of reagents. Indeed, technological solutions, such as cartridges for CL reagents handling or dissolvable walls for fluids flow control, have been often implemented to retain POC applicability [14,15]. To date, only a few origami CL µPADs exploiting smartphone detection have been reported, most of them relying on immunoassays [16,17,18]. On the other hand, several analytes of biochemical and diagnostic interest can be conveniently measured by enzyme-based biosensors, which take advantage of the high catalytic activity and high substrate selectivity of enzymes to provide sensitive and specific analyte detection in biological matrices [19,20].

We recently described the first foldable smartphone-based CL µPAD that exploited coupled enzyme reactions to perform on-site detection of organophosphorus compounds in environmental samples [21]. Nevertheless, this biosensor still presented some limitations partially impairing its POC applicability, such as an analytical protocol requiring sequential addition of reagents, relatively long assay time, and limited biosensor shelf-life. Herein, we describe a smartphone-based origami CL µPAD with improved features for simple and rapid quantitative glucose detection in biological samples at the POC. Indeed, by exploiting the origami architecture, a very simple and rapid two-step analytical protocol could be developed. Different from the previous biosensor [21], all the reagents were preloaded in the µPAD, so that the analysis required only the addition of the samples and a buffer solution, and no chemicals manipulation was required from the operator. Furthermore, reagents and assay procedures were optimized to provide reliable results and satisfactory long-term storage stability at room temperature of the biosensor, thus eliminating the need for controlled-temperature storage. Finally, a portable platform for coupling the µPAD with the smartphone’s CMOS camera was developed to enable easy and accurate real-time measurement of the flash CL signal, which is crucial for the applicability of the biosensor in POC settings.

## 2. Materials and Methods

### 2.1. Chemicals

Glucose oxidase (GOx) (EC 1.1.3.4, from *Aspergillus niger*, specific activity 210 U mg^−1^), potassium hexacyanoferrate(III), luminol (5-amino-2,3-dihydro-1,4-phthalazinedione) sodium salt, hydrogen peroxide solution 30% (*w*/*w*), glucose, pullulan (from *Aureobasidium pullulans*) and Whatman CHR 1 chromatographic paper (20 × 20 cm^2^ sheets) were purchased from Merck KGaA (Darmstadt, Germany). All other reagents were of the highest purity available.

Potassium hexacyanoferrate(III) (0.02 mol L^−1^) and luminol (0.2 mol L^−1^) solutions for the fabrication of the origami µPAD were prepared in deionized water and stored at 4 °C in the dark until use. Stock solutions of GOx (1000 U mL^−1^) were prepared in 0.1 mol L^−1^ phosphate buffer (pH 5.5) and stored at −20 °C until use. For loading in the biosensor, the GOx stock solution was diluted to the working concentration (50 U mL^−1^) with 0.01 mol L^−1^ phosphate buffer (pH 5.5) and supplemented by 50 mg mL^−1^ of pullulan as a stabilizing agent. Hydrogen peroxide and glucose standard solutions were prepared in 0.01 mol L^−1^ phosphate buffer (pH 5.5).

An enzymatic colorimetric assay in the 96-well microplate format (Invitrogen Glucose Colorimetric Detection Kit, Thermo Fisher Scientific, Waltham, MA, USA) was used as a reference method for assessing glucose concentration of real samples.

### 2.2. Samples

Assay performance has been tested by using commercially available quality control samples for clinical chemistry serum testing procedures (Lyphochek^®^ Assayed Chemistry Control, Bio-Rad Laboratories, Irvine, CA, USA).

### 2.3. Origami Paper Biosensor

**Figure 1:**
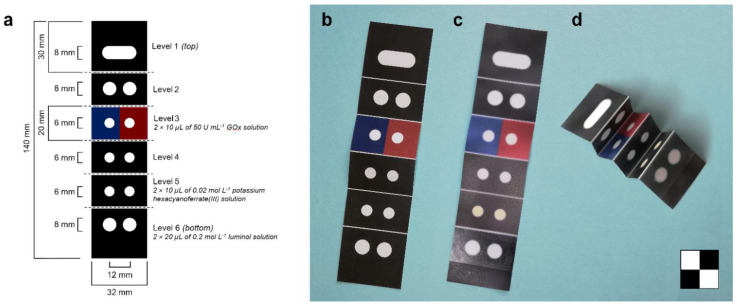
(**a**) Printing scheme of the origami µPAD with indication of the preloaded reagents (the red and blue areas indicate the loading positions of the sample and of the glucose standard solution, respectively). The dashed segments indicate the fold lines; (**b**) origami µPAD before heating to generate hydrophobic areas; (**c**) origami µPAD after heating, with preloaded reagents and applied clear adhesive tape; (**d**) origami µPAD folded for insertion into the biosensor holder. The chessboard is 2 × 2 cm^2^.

The origami µPAD (Figure 1) consisted of a 140 × 32 mm^2^ chromatographic paper sheet in which wettable hydrophilic areas are delimited by hydrophobic barriers obtained by wax printing.

To produce the biosensor, the pattern of the hydrophobic barriers (Figure 1a) was printed on a 20 × 20 cm^2^ Whatman CHR 1 chromatography paper sheet using a commercial solid wax Phaser 8560DN printer (Xerox Co., Norwalk, CT, USA). The printed sheet was heated to 100 °C in an oven for 5 min to melt the wax and generate the hydrophobic barriers. Fold lines were created using a manual rotary perforating blade to facilitate the folding of the biosensor. Then, the sheet was cut to obtain the single origami biosensors (five biosensors can be printed on a 20 × 20 cm^2^ paper sheet). The reagents were loaded into the origami µPAD by dispensing their solutions into the two hydrophilic areas of levels 3 (2 × 10 µL of 50 U mL^−1^ GOx solution), 5 (2 × 10 µL of 0.02 mol L^−1^ potassium hexacyanoferrate(III) solution), and 6 (2 × 20 µL of 0.2 mol L^−1^ luminol solution). After air-drying at room temperature in the dark for 1 h, a clear adhesive tape was applied to level 6 of the biosensor to prevent the carbonate transport buffer from spilling into the device during the assay. For long-term storage, the biosensors were vacuum-sealed in polyethylene bags and kept in the dark until use.

### 2.4. Assay Device

**Figure 2:**
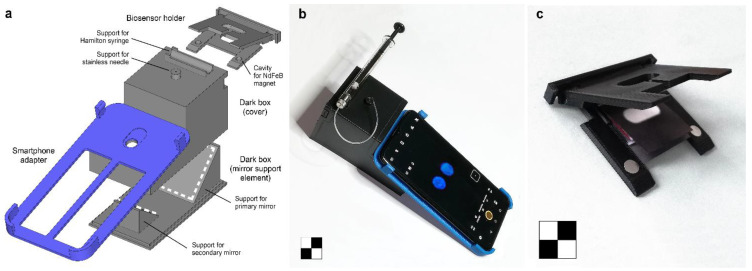
(**a**) Schematic drawing of the 3D-printed elements of the analytical device. The white dashed lines indicate the positions of the two mirrors; (**b**) analytical device connected to the OnePlus 6 smartphone; (**c**) biosensor holder with an origami µPAD inserted. The chessboards are 2 × 2 cm^2^.

The assay device components (Figure 2a) were designed using SketchUp Pro 2021 CAD software (Trimble Inc., Sunnyvale, CA, USA) and manufactured from acrylonitrile-butadiene-styrene (ABS) polymer using a commercial fused deposition modeling (FDM) 3D printer (MakerBot Replicator 2X, MakerBot Industries, New York, NY, USA). Except for the smartphone adapter, they have been made of black ABS to avoid interference from ambient light as well as unwanted light reflections inside the device during measurement. The overall dimensions of the device (without the smartphone adapter, the dimensions of which may vary depending on the smartphone model) were 60 mm (W) × 60 mm (H) × 110 mm (D).

In more detail, the 3D printed components of the assay device were as follows:

- A biosensor holder keeping the origami µPAD neatly folded during the assay to ensure homogeneous migration of the carbonate transport buffer. Four small NdFeB magnets (N45 grade, 5 mm diameter, 1 mm height) were embedded into the two halves of the holder to apply pressure on the folded biosensor, and the upper and lower openings allowed delivery of the transport buffer and imaging of the CL emission, respectively;
- A dark box consisting of a mirror support element with two flat mirrors (a primary 40 × 40 mm^2^ mirror and a secondary 25 × 25 mm^2^ one) angled at approximately 45°, which allowed the origami biosensor to be imaged from below, and a cover to support the smartphone adapter and to avoid interference from ambient light during the measurement. Mirrors (first surface mirrors with protected silver coating, purchased from Edmund Optics, Ltd., York, UK) have a reflectivity above 98% in the 450–2000 nm wavelength range. For the measurement, the cover accepted the biosensor holder and a stainless-steel needle connected with a silicone rubber tube (0.5 mm I.D.) to a 1000 µL Hamilton syringe for dispensing the carbonate transport buffer during the assay;
- A smartphone adapter specifically designed to fit a OnePlus 6 smartphone (OnePlus, Shenzen, China).

Figure 2b,c show the device connected to the OnePlus 6 smartphone and the biosensor holder with an origami µPAD inserted.

### 2.5. Assay Procedure

The origami µPAD was removed from the sealed plastic bag. Ten µL of a serum sample, previously diluted 1:50 (*v*/*v*) with 0.01 mol L^−1^ phosphate buffer (pH 5.5), and of a 100 µmol L^−1^ glucose standard solution were deposited in the sample and standard loading positions of the biosensor, respectively. After 10 min of incubation at room temperature, the biosensor was folded, inserted into the holder, and placed in the dark box of the device. Before starting the assay, a background correction image was acquired using an exposure time of 15 s and ISO 800 sensitivity. Then, a sequence of 10 consecutive image acquisitions was started using the same acquisition parameters. Immediately after the start of the image acquisition, 200 µL of 0.1 mol L^−1^ carbonate buffer (pH 10.0) were rapidly dispensed onto the origami µPAD using the Hamilton syringe connected to the dark box. Image capture was performed using the Android Camera FV-5 app, available on Google Play (other camera apps enabling long exposure times and automated acquisition of image sequences could also be used). The CL images were analyzed using the freeware ImageJ v.1.53h software (National Institutes of Health, Bethesda, MD, USA). First, regions of interest (ROIs) corresponding to the hydrophilic areas of level 6 of the biosensor were defined, and for each image the CL signals were evaluated by integrating the CL emissions on the ROI areas. Then, after subtracting the CMOS sensor background signal measured in the background correction image, the total CL signals for the sample and the glucose standard solution were calculated by summing the CL signals of all images. Finally, the glucose concentration of the sample was calculated using the following equation:

(1) $$\mathrm{Glucose}\;\mathrm{concentration}=\frac{\mathrm{sample}\;\mathrm{CL}\;\mathrm{signal}}{\mathrm{standard}\;\mathrm{CL}\;\mathrm{signal}}\times 100{\;\mu \mathrm{mol}\;L}^{-1}\times \mathrm{sample}\;\mathrm{dilution}\;\mathrm{factor}.$$

## 3. Results and Discussion

### 3.1. Design of the Origami µPAD and of the Analytical Device

Origami paper-based devices have offered researchers new opportunities for implementing complex multistep analytical procedures in µPADs. We used the origami approach in designing a µPAD for rapid and easy assessment of blood glucose levels. It consisted of a sheet of chromatographic paper with hydrophilic areas delimited by wax-printed hydrophobic barriers (Figure 1). Once folded, the biosensor consisted of six levels, from level 1 (top) to level 6 (bottom), each with a specific role. Levels 1 and 2 evenly distributed the flow of the transport buffer (which is delivered at level 1) to the lower levels of the biosensor. Levels 3, 5, and 6 contained the reagents required for the assay, namely GOx (level 3), potassium hexacyanoferrate(III) (level 5), and luminol (level 6). Level 4 had the role of preventing premature contact between the hydrogen peroxide produced in level 3 and the CL reagents in the lower levels when the biosensor is folded before insertion into the holder. The origami µPAD featured a dual assay design since each level (except level 1) had two separate hydrophilic areas so that a sample and a glucose standard solution could be simultaneously measured. The analytical procedure involved two steps. The first one, performed in the unfolded biosensor, consisted of the addition of the sample and glucose standard solutions to level 3 of the biosensor, followed by an incubation period to achieve the complete GOx-catalyzed oxidation of glucose with the production of D-glucono-δ-lactone and hydrogen peroxide. Then, the origami µPAD was folded to stack the hydrophilic areas, and a carbonate transport buffer was dispensed on the top level of the origami. The buffer migrated by capillarity toward the lower levels of the biosensor carrying the hydrogen peroxide produced in level 3 and dissolving the CL reagents loaded in levels 5 and 6. It also provided the alkaline pH necessary for the CL reaction. This resulted in the production of CL signals in the lowest level of the biosensor, the intensity of which was proportional to the amount of glucose in the sample and in the glucose standard solution. Finally, the concentration of glucose in the sample was assessed by comparing the two CL signals.

An analytical device was designed to perform smartphone-based analysis at the POC (Figure 2). It included a smartphone adapter designed to image the CL signal through the primary 16-megapixel camera of a OnePlus 6 smartphone. However, to increase the versatility of the device, the adapter was an independent component and could easily be replaced with a different one so that other smartphones could be used with minimal effort. Almost all smartphones currently on the market have multi-camera configurations, so the most suitable camera for CL imaging must be chosen for each model. In modern smartphones, the primary cameras often exceed 50 megapixels, but high image resolution is not a requirement for this device. Indeed, lower resolution cameras may use CMOS sensors with larger pixels, which is an advantage when acquiring weak light signals (i.e., higher signal-to-noise ratios can be achieved). In addition, it is important that all automatic image enhancement features are disabled as they may alter images, thus hindering accurate quantification of the CL signal.

Compared to other optical sensing principles, such as colorimetry and fluorescence, CL sensing requires a simpler instrument setup since no sample illumination systems, specific sample geometries, excitation light sources, or wavelength selection systems are required. Therefore, the compactness of the analytical device depended mainly on the minimum distance required between the smartphone and the sample imaging area. Smartphones can focus on objects a few centimeters away, and additional lenses could further reduce the focus distance [22]. However, at short distances, it is not possible to image large areas, or, at best, the perspective distortion of the images could affect the measurement. As previously described [23], we used two flat angled mirrors to fold the path of the CL emission and thus increase the distance between the smartphone’s CMOS camera and the detection zone without compromising the compactness of the device. The use of mirrors also made it possible to image the CL signal generated in the lowest level of the biosensor from below. In this way, the orientation of the biosensor allowed easy addition of the transport buffer, and the acquisition of the CL images was performed by keeping the smartphone with the touchscreen facing upwards, i.e., in a suitable position to operate the controls of the camera and monitor the image capture process. Previously reported architectures for coupling CL paper-based biosensors with smartphone detection were not suitable for recording flash-type CL reactions, thus limiting the applicability of this approach [21].

### 3.2. Optimization of Experimental Parameters: Detection of Hydrogen Peroxide

**Figure 3:**
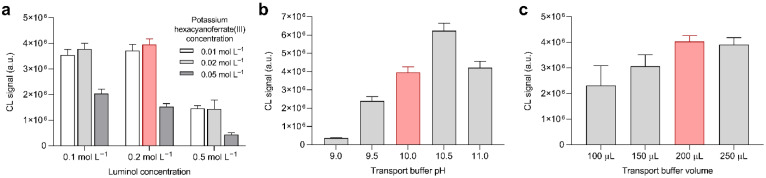
Optimization of experimental conditions for hydrogen peroxide detection, performed by analyzing 10 µL of a 100 µmol L^−1^ hydrogen peroxide standard solution (the selected conditions are highlighted). (**a**) CL signals obtained in origami µPADs containing different amounts of luminol and potassium hexacyanoferrate(III); (**b**) CL signals obtained with carbonate transport buffers at different pH; (**c**) CL signals obtained using different carbonate transport buffer volumes. In each measurement, the optimal values for the other experimental conditions were used. Each value is the mean ± SD of six measures performed in three origami µPADs.

First, the CL detection of hydrogen peroxide was optimized by analyzing a 100 µmol L^−1^ hydrogen peroxide standard solution employing origami µPADs that contained different amounts of potassium hexacyanoferrate(III) and luminol preloaded in levels 5 and 6 (overall CL signal integration time 150 s, see below). The procedure used was similar to that employed for serum samples, except that the origami µPADs did not contain GOx and the hydrogen peroxide solutions (dispensed, as for blood samples, on level 3 of the biosensor) were immediately analyzed without any preliminary incubation period. Furthermore, no comparison with glucose standard solution was needed; thus, two samples could be simultaneously assayed. As shown in Figure 3a, the strongest CL signal was obtained in biosensors prepared using 0.02 mol L^−1^ potassium hexacyanoferrate(III) and 0.2 mol L^−1^ luminol solutions, so these values were adopted for the biosensor. The CL reaction of hydrogen peroxide with the hexacyanoferrate(III)/luminol system required an alkaline pH, which was provided by the carbonate transport buffer. A high concentration of the buffer (0.1 mol L^−1^) was employed to ensure that its pH was not altered by mixing with the sample or by the dissolution of the dry reagents preloaded in the device. We measured the intensity of the CL signal for pH values ranging from 9.0 to 11.0, obtaining the most intense CL emission at pH 10.5 (Figure 3b). However, since the CL signal of the blank (due to the reaction of luminol with atmospheric oxygen) was also strongly pH-dependent, the highest signal to blank ratio (ca. 40 for the 100 µmol L^−1^ hydrogen peroxide concentration) was at pH 10.0. This result is consistent with what was reported by Warm et al., who obtained the best signal to blank ratio at pH 9.5 in a test tube-based assay based on the luminol/hexacyanoferrate(III) CL system [24]. The volume of the carbonate transport buffer was also considered. Figure 3c indicated that 200 μL of buffer was sufficient to transport the hydrogen peroxide to the lower levels of the origami biosensor and dissolve the dry reagents preloaded in those levels. Higher volumes of buffer were not used to avoid the risk of buffer leaking into the device, while smaller ones provided weaker and less reproducible CL signals, suggesting both an incomplete transport of hydrogen peroxide and an uneven distribution of the buffer within the biosensor (in some cases it was observed that the hydrophilic areas of level 6 were not completely wet).

**Figure 4:**
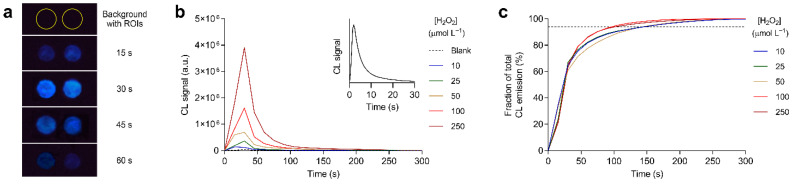
(**a**) Background correction image and first CL images acquired during an assay. The ROIs used for quantification of the CL signal are shown in the background correction image; (**b**) kinetic profiles of the CL signals obtained in the origami µPAD for different concentrations of hydrogen peroxide (time zero corresponds to the addition of the carbonate transport buffer). The inset shows the kinetic profile of the CL signal observed in solution after mixing luminol, potassium hexacyanoferrate(III), and hydrogen peroxide; (**c**) fraction of the total CL emission as a function of the time elapsed since the delivery of the carbonate transport buffer for different concentrations of hydrogen peroxide. The dashed line corresponds to 95% of the total CL emission.

The CL emission kinetics was also studied to define the assay procedure (Figure 4a). Figure 4b shows the kinetics of CL emissions recorded for hydrogen peroxide standard solutions ranging from 0 to 250 µmol L^−1^. The kinetics was flash type, as also observed in solution (see inset of Figure 4b), but the onset and decay of emissions were slower, and the maximum CL signal was obtained between 15 and 30 s after the addition of the carbonate transport buffer, while in solution the CL emission peak was reached in a few seconds. This was consistent with the fact that the CL reaction in the biosensor was mainly controlled by the flow of the carbonate transport buffer and by the dissolution of the dry CL reagents, rather than by the diffusion of reagents in solution. The CL signal disappeared 5 min after the addition of the carbonate transport buffer, but most of the emission occurred in shorter times. In fact, at least 95% of the emission was produced within 150 s from the addition of the buffer (Figure 4c), so this time was chosen as the integration time for the evaluation of the CL signal. Furthermore, the measurements confirmed that the nonspecific CL signal obtained in the absence of hydrogen peroxide was very weak, which would avoid the need for a blank analysis during the assay.

**Figure 5:**
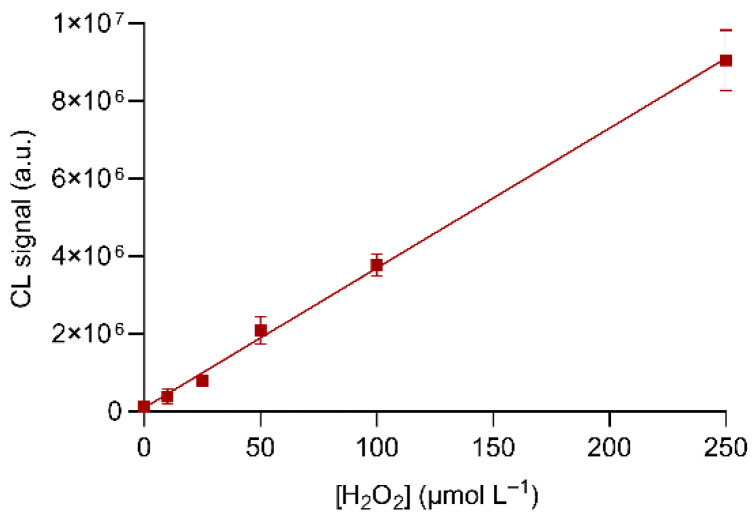
Calibration curve for hydrogen peroxide obtained in the optimized experimental conditions. The equation of the calibration curve is Y = (3.60 × 10^4^ ± 2.0 × 10^3^)X + (9.4 × 10^4^ ± 1.7 × 10^5^), R^2^ = 0.993, where Y is the CL signal and X is the concentration of hydrogen peroxide (µmol L^−1^). Each value is the mean ± SD of six measures performed in three origami µPADs.

The analytical performance of the origami µPAD for detecting hydrogen peroxide was evaluated by generating a calibration curve in the concentration range 0–250 µmol L^−1^. The calibration curve (Figure 5) showed a suitable linear correlation (R^2^ > 0.99) between the CL signal and the amount of hydrogen peroxide in the whole concentration range. The limit of detection (LOD) of the assay, estimated as the concentration of hydrogen peroxide corresponding to the CL signal of the blank plus three times its standard deviation, was 4.0 µmol L^−1^, which corresponded to 40 pmol of hydrogen peroxide. This value is comparable with those of other paper-based assays for hydrogen peroxide detection [25,26,27] and lower than that reported by Warm et al. [24]. We expected a similar LOD value for glucose, considering that the glucose oxidation reaction produces hydrogen peroxide in the 1:1 stoichiometric ratio.

### 3.3. Optimization of Experimental Parameters: Detection of Glucose

**Figure 6:**
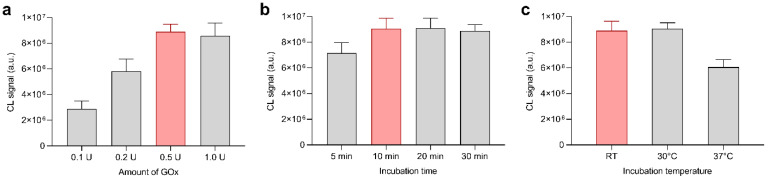
Optimization of experimental conditions for glucose detection, performed by analyzing 10 µL of a 250 µmol L^−1^ glucose standard solution (the selected conditions are highlighted). (**a**) CL signals obtained in origami µPADs containing different amounts of GOx. (**b**) CL signals obtained with different incubation times. (**c**) CL signals obtained with different incubation temperatures. In each measurement, the optimal values for the other experimental conditions were used. Room temperature (RT) varied between 20 and 25 °C. Each value is the mean ± SD of six measures performed in three origami µPADs.

Then, the experimental parameters for the GOx-catalyzed glucose oxidation were optimized to ensure complete glucose conversion in the glucose calibration range (i.e., up to 250 µmol L^−1^). Indeed, an endpoint assay is more reliable as it is not affected by factors such as decrease in enzymatic activity due, for example, to the aging of the biosensor or to sub-optimal storage conditions. According to the literature [28], the maximum activity of GOx from *Aspergillus niger* was at pH 5.5; therefore, this pH was adopted for samples and glucose standard solutions. We investigated the influence of both the amount of enzyme loaded into the origami µPAD and the incubation time. Figure 6a shows the dependence of the CL signal on the activity of the GOx preloaded in the biosensor. The CL signal increased with the amount of enzyme up to 0.5 U and then remained constant, suggesting that 0.5 U of the enzyme was sufficient to achieve a complete glucose conversion. As expected, a similar trend was observed for the incubation time (Figure 6b), for which the maximum CL signal was reached after 10 min of incubation. Regarding the incubation temperature, Figure 6c shows that a controlled-temperature environment was not required, thus simplifying the assay procedure. Indeed, the glucose conversion was complete even at room temperature (in our experiments, ambient temperature varied between 20 and 25 °C). Surprisingly, although the rate of the enzyme-catalyzed reaction is expected to increase with temperature and the optimal temperature range for GOx activity extends up to 40 °C [29], the highest incubation temperature (i.e., 37 °C) resulted in the weakest CL signal. We assumed that the lower CL signal might be due to the partial evaporation of water (sample volume is only 10 µL), which affected the enzyme reaction. In the preparation of the µPAD, we also considered its stability upon storage, considering that room temperature would be preferred for POC settings. To reach this goal, pullulan, a polysaccharide produced by the fungus *Aureobasidium pullulans*, was added to the GOx solution to extend the enzyme stability [30].

**Figure 7:**
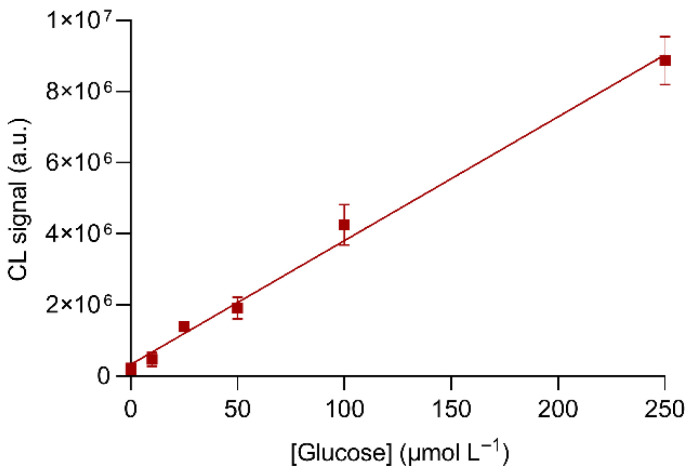
Calibration curve for glucose obtained in the optimized experimental conditions. The equation of the calibration curve is Y = (3.51 × 10^4^ ± 2.5 × 10^3^)X + (3.3 × 10^5^ ± 3.9 × 10^5^), R^2^ = 0.989, where Y is the CL signal and X is the concentration of glucose (µmol L^−1^). Each value is the mean ± SD of six measures performed in three origami µPADs.

The analytical performance of the origami µPAD for detecting glucose was evaluated by generating a calibration curve for glucose concentrations up to 250 µmol L^−1^. The calibration curve (Figure 7) showed suitable linearity (R^2^ > 0.98) with a LOD value of about 10 µmol L^−1^, corresponding to 100 pmol of glucose. The slope of the calibration curve was close to that obtained for hydrogen peroxide, again supporting the hypothesis that in the optimized experimental conditions, the GOx-catalyzed oxidation of glucose was complete.

To the best of our knowledge, only a very few paper-based CL glucose biosensors have been reported in the literature, and they displayed LODs similar or higher with respect to our system. Li et al. recently described a 3D paper-based μPAD for the detection of glucose, lactate, cholesterol, and choline, exploiting the oxidation of each analyte catalyzed by a specific oxidase followed by the CL detection of hydrogen peroxide [20]. The CL measurements were carried on an ultraweak luminescence analyzer, and the low LOD values (e.g., 8 μmol L^−1^ for glucose) allowed to measure the target analytes in diluted serum samples. The wax screen-printing technology was used to develop a low-cost, cloth-based CL biosensor able to measure glucose in the range 0.01–10 mmol L^−1^ and with a LOD of 9.07 μmol L^−1^ by exploiting CCD-based imaging detection [31]. A CL μPAD for the simultaneous detection of glucose and uric acid by means of GOx and urate oxidase was also described by Yu et al. in 2011 [32]. However, the LOD for glucose of this device was relatively high (140 μmol L^−1^) even though a sensitive photomultiplier-based CL detector was used. Other luminescence-based μPADs for glucose described in the literature rely on electrochemiluminescence (ECL). They provided tens of μmol L^−1^ [33] or, in some cases, sub-μmol L^−1^ detection limits [34], but the procedure for obtaining μPADs equipped with electrodes is complex, and additional hardware is needed to produce and control the potential driving the ECL emission.

### 3.4. Biosensor Stability

**Figure 8:**
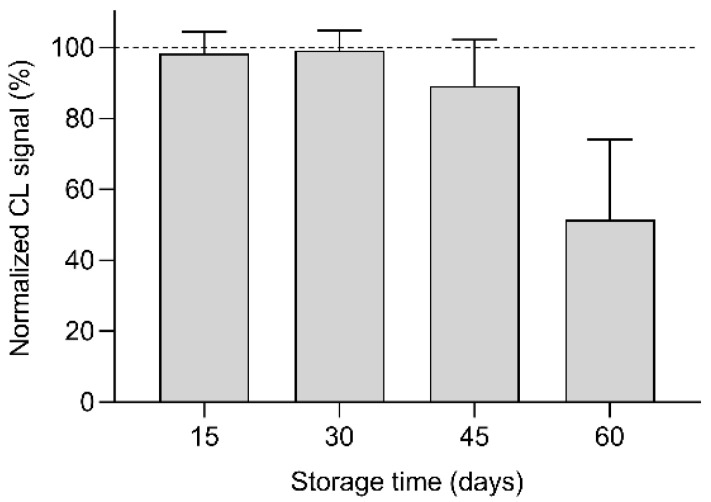
Changes of the CL signals obtained by analyzing 10 µL of a 250 µmol L^−1^ glucose standard solution using origami µPADs stored in vacuum-sealed polyethylene bags at room temperature and in the dark for different times. The CL signals were normalized to that obtained using the origami µPADs just after their production. Each value is the mean ± SD of six measures performed in five origami µPADs.

The stability over time of the origami µPAD was evaluated using a series of µPADs prepared using the same reagent batches, vacuum-sealed in polyethylene bags, and stored at room temperature in the dark until use. The decrease in the performance during storage was evaluated by analyzing a 250 µmol L^−1^ glucose standard solution at different storage times and comparing the CL signals with that obtained using the origami µPAD just after their production. According to the results shown in Figure 8, the µPAD performance remains unchanged for up to 30 days of storage.

### 3.5. Analysis of Real Samples

By exploiting the dual assay design of the origami µPAD, we have developed an analytical procedure that does not require the generation of a calibration curve (which would require additional time) nor the use of a stored one (whose validity must be verified prior to the assay). Thanks to the linear correlation between the CL signal and the glucose concentration and the low blank signal, we evaluated the concentration of a sample by simultaneously analyzing a 100 µmol L^−1^ glucose standard solution and comparing the CL signals obtained according to Equation (1). This procedure allowed to perform the analysis in a single run (assay time was less than 20 min). It also reduced the effect of external factors affecting the CL signal intensity (e.g., ambient temperature), which is of relevance for portable analytical devices as they operate in conditions where it is difficult to control environmental parameters.

Blood glucose levels in fasting healthy subjects are in the range 70–130 mg dL^−1^ (corresponding to 3.9–7.1 mmol L^−1^) [35], therefore, to comply with the calibration interval of the µPAD (which extended up to 250 µmol L^−1^) samples were diluted 1:50 (*v*/*v*) with 0.01 mol L^−1^ phosphate buffer (pH 5.5). Considering this preliminary dilution, the concentrations of normal blood samples analyzed with the biosensor will range between 80 and 140 µmol L^−1^, so both hyperglycemic and hypoglycemic samples could be discriminated. Furthermore, the high dilution factor avoids interference due to the sample matrix.

To evaluate assay applicability on real samples, we employed quality control serum samples spiked with known amounts of glucose after reconstitution according to the vendor’s instruction. For reference, their glucose concentration was also measured with a commercial enzymatic colorimetric glucose assay for biological samples. As shown in Table 1, there is a suitable correspondence between the glucose concentrations measured with the origami µPAD and the reference assay (bias values ranged between +4.3% and −5.1%), which suggested the applicability of the biosensor for assessing glucose blood levels. The biosensor also showed satisfactory reproducibility, with RSD values lower than 6.5% (N = 5) for the assayed concentration levels.

## 4. Conclusions

This article describes an origami µPAD for the quantitative determination of glucose in blood samples based on its oxidation by glucose oxidase followed by the detection of hydrogen peroxide by the CL luminol/hexacyanoferrate (III) system. By taking advantage of our previous experience [21], a simple and rapid CL biosensor displaying improved performance for POC application was developed. The origami approach made it possible to implement a two-step analytical procedure and to avoid chemical handling by the operator, as all the reagents were preloaded in the biosensor. To facilitate the analysis in POC settings, reagents formulation was optimized to allow biosensor storage at room temperature, and a portable analytical device was produced by 3D printing for measuring the CL emission using a smartphone. The assay proved suitable for the detection of glucose in the blood, allowing to discriminate both hypoglycemic and hyperglycemic samples in a short time (i.e., less than 20 min). The same approach could be used for other clinically relevant analytes, such as uric acid or lactate, which are relatively abundant and can be determined using specific oxidases.

## Figures and Tables

**Table 1:** Comparison of glucose concentrations measured in non-spiked and glucose-spiked control samples with the origami µPAD and the reference colorimetric assay.

Lyphochek^®^ Assayed Chemistry Control	Added Glucose (mmol L^−1^)	Measured Glucose (mmol L^−1^) ^1^		Bias (%)
		Reference Colorimetric Assay	Origami µPAD	
Level 1	0.0	5.11 ± 0.15	5.33 ± 0.28	+4.3
	2.0	7.07 ± 0.14	6.95 ± 0.39	−1.7
	4.0	9.18 ± 0.14	9.33 ± 0.35	+1.6
	6.0	11.12 ± 0.28	11.19 ± 0.53	+0.6
	8.0	13.06 ± 0.23	12.38 ± 0.78	−5.2
Level 2	0.0	16.53 ± 0.44	^2^	n.d.

^1^ Calculated upon correction for dilution factor. Each value is the mean ± SD of five measures. ^2^ Out of origami µPAD calibration range.

## Data Availability

Not applicable.

## References

[1] Land K.J., Boeras D.I., Chen X.S., Ramsay A.R., Peeling R.W. (2019). REASSURED diagnostics to inform disease control strategies, strengthen health systems and improve patient outcomes. Nat. Microbiol..

[2] Lim H., Jafry A.T., Lee J. (2019). Fabrication, flow control, and applications of microfluidic paper-based analytical devices. Molecules.

[3] Ozer T., McMahon C., Henry C.S. (2020). Advances in paper-based analytical devices. Annu. Rev. Anal. Chem..

[4] Suntornsuk W., Suntornsuk L. (2020). Recent applications of paper-based point-of-care devices for biomarker detection. Electrophoresis.

[5] Puiu M., Mirceski V., Bala C. (2021). Paper-based diagnostic platforms and devices. Curr. Opin. Electrochem..

[6] Lee W.C., Ng H.Y., Hou C.Y., Lee C.T., Fu L.M. (2021). Recent advances in lab-on-paper diagnostic devices using blood samples. Lab. Chip.

[7] Singh A.T., Lantigua D., Meka A., Taing S., Pandher M., Camci-Unal G. (2018). Paper-based sensors: Emerging themes and applications. Sensors.

[8] Huang X.W., Xu D.D., Chen J., Liu J.X., Li Y.B., Song J., Ma X., Guo J.H. (2018). Smartphone-based analytical biosensors. Analyst.

[9] Geng Z.X., Zhang X., Fan Z.Y., Lv X.Q., Su Y., Chen H.D. (2017). Recent progress in optical biosensors based on smartphone platforms. Sensors.

[10] Ge L., Yu J.H., Ge S.G., Yan M. (2014). Lab-on-paper-based devices using chemiluminescence and electrogenerated chemiluminescence detection. Anal. Bioanal. Chem..

[11] Mirasoli M., Guardigli M., Michelini E., Roda A. (2014). Recent advancements in chemical luminescence-based lab-on-chip and microfluidic platforms for bioanalysis. J. Pharm. Biomed. Anal..

[12] Roda A., Arduini F., Mirasoli M., Zangheri M., Fabiani L., Colozza N., Marchegiani E., Simoni P., Moscone D. (2020). A challenge in biosensors: Is it better to measure a photon or an electron for ultrasensitive detection?. Biosens. Bioelectron..

[13] Calabretta M.M., Zangheri M., Calabria D., Lopreside A., Montali L., Marchegiani E., Trozzi I., Guardigli M., Mirasoli M., Michelini E. (2021). Paper-based immunosensors with bio-chemiluminescence detection. Sensors.

[14] Chu W.R., Chen Y., Liu W., Zhao M., Li H.F. (2017). Paper-based chemiluminescence immunodevice with temporal controls of reagent transport technique. Sens. Actuator B-Chem..

[15] Zangheri M., Mirasoli M., Guardigli M., Di Nardo F., Anfossi L., Baggiani C., Simoni P., Benassai M., Roda A. (2019). Chemiluminescence-based biosensor for monitoring astronauts’ health status during space missions: Results from the International Space Station. Biosens. Bioelectron..

[16] Ge L., Wang S.M., Song X.R., Ge S.G., Yu J.H. (2012). 3D Origami-based multifunction-integrated immunodevice: Low-cost and multiplexed sandwich chemiluminescence immunoassay on microfluidic paper-based analytical device. Lab. Chip.

[17] Liu W., Cassano C.L., Xu X., Fan Z.H. (2013). Laminated paper-based analytical devices (LPAD) with origami-enabled chemiluminescence immunoassay for cotinine detection in mouse serum. Anal. Chem..

[18] Roda A., Zangheri M., Calabria D., Mirasoli M., Caliceti C., Quintavalla A., Lombardo M., Trombini C., Simoni P. (2019). A simple smartphone-based thermochemiluminescent immunosensor for valproic acid detection using 1,2-dioxetane analogue-doped nanoparticles as a label. Sens. Actuat. B-Chem..

[19] Yu J.H., Wang S.M., Ge L., Ge S.G. (2011). A novel chemiluminescence paper microfluidic biosensor based on enzymatic reaction for uric acid determination. Biosens. Bioelectron..

[20] Li F., Liu J.C., Guo L., Wang J.H., Zhang K.Q., He J.B., Cui H. (2019). High-resolution temporally resolved chemiluminescence based on double-layered 3D microfluidic paper-based device for multiplexed analysis. Biosens. Bioelectron..

[21] Montali L., Calabretta M.M., Lopreside A., D’Elia M., Guardigli M., Michelini E. (2020). Multienzyme chemiluminescent foldable biosensor for on-site detection of acetylcholinesterase inhibitors. Biosens. Bioelectron..

[22] Calabria D., Mirasoli M., Guardigli M., Simoni P., Zangheri M., Severi P., Caliceti C., Roda A. (2020). Paper-based smartphone chemosensor for reflectometric on-site total polyphenols quantification in olive oil. Sens. Actuat. B-Chem..

[23] Calabria D., Guardigli M., Severi P., Trozzi I., Pace A., Cinti S., Zangheri M., Mirasoli M. (2021). A smartphone-based chemosensor to evaluate antioxidants in agri-food matrices by in situ AuNP formation. Sensors.

[24] Warm E., Laties G.G. (1982). Quantification of hydrogen peroxide in plant extracts by the chemiluminescence reaction with luminol. Phytochemistry.

[25] Zhang W.C., Niu X.H., Li X., He Y.F., Song H.W., Peng Y.X., Pan J.M., Qiu F.X., Zhao H.L., Lan M.B. (2018). A smartphone-integrated ready-to-use paper-based sensor with mesoporous carbon-dispersed Pd nanoparticles as a highly active peroxidase mimic for H_2_O_2_ detection. Sens. Actuat. B-Chem..

[26] Ragavan K.V., Ahmed S.R., Weng X., Neethirajan S. (2018). Chitosan as a peroxidase mimic: Paper based sensor for the detection of hydrogen peroxide. Sens. Actuat. B-Chem..

[27] Sanchez-Calvo A., Costa-Garcia A., Blanco-Lopez M.C. (2020). Paper-based electrodes modified with cobalt phthalocyanine colloid for the determination of hydrogen peroxide and glucose. Analyst.

[28] Weibel M.K., Bright H.J. (1971). The glucose oxidase mechanism: Interpretation of the pH dependence. J. Biol. Chem..

[29] Gouda M.D., Singh S.A., Rao A.G.A., Thakur M.S., Karanth N.G. (2003). Thermal inactivation of glucose oxidase. Mechanism and stabilization using additives. J. Biol. Chem..

[30] Jahanshahi-Anbuhi S., Kannan B., Leung V., Pennings K., Liu M., Carrasquilla C., White D., Li Y.F., Pelton R.H., Brennan J.D. (2016). Simple and ultrastable all-inclusive pullulan tablets for challenging bioassays. Chem. Sci..

[31] Li H.J., Wang D., Liu C.L., Liu R., Zhang C.S. (2017). Facile and sensitive chemiluminescence detection of H_2_O_2_ and glucose by a gravity/capillary flow and cloth-based low-cost platform. RSC Adv..

[32] Yu J.H., Ge L., Huang J.D., Wang S.M., Ge S.G. (2011). Microfluidic paper-based chemiluminescence biosensor for simultaneous determination of glucose and uric acid. Lab. Chip.

[33] Chen L., Zhang C.S., Xing D. (2016). Paper-based bipolar electrode-electrochemiluminescence (BPE-ECL) device with battery energy supply and smartphone read-out: A handheld ECL system for biochemical analysis at the point-of-care level. Sens. Actuat. B-Chem..

[34] Wang D., Liang Y., Su Y., Shang Q.P., Zhang C.S. (2019). Sensitivity enhancement of cloth-based closed bipolar electrochemiluminescence glucose sensor via electrode decoration with chitosan/multi-walled carbon nanotubes/graphene quantum dots-gold nanoparticles. Biosens. Bioelectron..

[35] Danaei G., Finucane M.M., Lu Y., Singh G.M., Cowan M.J., Paciorek C.J., Lin J.K., Farzadfar F., Khang Y.H., Stevens G.A. (2011). National, regional, and global trends in fasting plasma glucose and diabetes prevalence since 1980: Systematic analysis of health examination surveys and epidemiological studies with 370 country-years and 2·7 million participants. Lancet.

